# Supplementing 25-Hydroxy-Vitamin D_3_ to Sows Enhances Milk and Blood Parameters, with Extended Benefits to Their Offspring

**DOI:** 10.3390/ani15223264

**Published:** 2025-11-11

**Authors:** Shanmugam Sureshkumar, Md Raihanul Hoque, In Ho Kim

**Affiliations:** 1 Department of Animal Biotechnology, Dankook University, Anseodong, Cheonan 31116, Republic of Korea; sureshbiogenetic@gmail.com (S.S.); raihan36507@gmail.com (M.R.H.); 2 Smart Animal Bio Institute, Dankook University, Cheonan 31116, Republic of Korea

**Keywords:** 25 hydroxy-vitamin D_3_, IgG, sow reproduction, milk composition

## Abstract

The objective of this study was to evaluate the effects of 25-hydroxy-vitamin D_3_ (25OHD_3_) supplementation on reproductive performance, nutrient digestibility, lameness score, milk composition, and blood profiles in sows, as well as the performance and blood profiles of their offspring. From day 110 of gestation to 21 days of weaning, a total of 30 multiparous sows (Landrace × Yorkshire) were randomly assigned to one of three dietary treatments, with ten sows per treatment. The dietary treatments were: (1) CON, basal diet; (2) TRT1, CON diet plus 1114 IU 25OHD_3_/kg (13.92 µg 25OHD_3_/kg feed); and (3) TRT2, CON diet plus 2227 IU 25OHD_3_/kg (27.84 µg 25OHD_3_/kg feed). The reproduction performance and nutrient digestibility of sows were not affected by 25OHD_3_ supplementation. However, the inclusion of graded levels of 25OHD_3_ in the sow diet had significantly reduced their farrowing time (*p* < 0.001) compared to those fed the CON diet. Also, sows fed 25OHD_3_ produced significantly higher (*p* < 0.05) number of piglets compared to the CON group. Piglets from supplemented sows exhibited greater (*p* < 0.05) average daily gain and weaning body weight. Moreover, colostrum protein content was higher (*p* < 0.05) in sows fed 25OHD_3_ compared with the CON group. Similarly, the concentration of 25OHD_3_ in colostrum and milk at weaning was markedly (*p* < 0.001) elevated. Furthermore, serum 25OHD_3_ levels were significantly higher (*p* < 0.05) in both sows and piglets, and piglet serum immunoglobulin G (IgG) concentrations were also elevated (*p* < 0.05) in the supplemented groups. In summary, dietary 25OHD_3_ in the sow diet not only improves their reproductive performance and milk quality but also enhances piglet growth, immunity, and overall vitality, suggesting that 25OHD_3_ is a valuable nutritional strategy for optimizing sow productivity and promoting healthier offspring.

## Simple Summary

Vitamin D is essential for maintaining bone strength, reproductive efficiency, and immune function. In sow diets, it supports healthy pregnancies, optimal milk production, and robust piglet growth. However, research on the use of 25-hydroxy-vitamin D_3_ (25OHD_3_), a more biologically active and efficient form of vitamin D in sow nutrition, remains limited. Therefore, we conducted this study to examine how adding 25OHD_3_ to sow diets could enhance their performance and the health status of their offspring. As expected, sow-fed diets containing 25OHD_3_ improved reproductive performance, enhanced milk quality, and boosted the growth and immune health of piglets. Therefore, we believe that these findings offer a new perspective on the use of 25OHD_3_ in sow diets and may serve as a practical approach to enhance overall pig production.

## 1. Introduction

Proper nutrition, careful management, and a periodical evaluation of reproduction performance are major procedures to maintain a successful breeding sow herd [1]. Reproductive problems are major reasons for early culling in sows [2]. Vitamin D sufficiency is important for the timely pubertal transition and maintenance of female reproductive function [3]. Moreover, the metabolic health of offspring mainly depends on maternal vitamin D status [4] as it can affect farrowing time, piglet birth weight, and mortality [5]. Vitamin D can help maintain proper development and function of the immune system [6]. The physiological status of vitamin D can suppress pro-inflammatory response and regulate the innate and adaptive immune system [7]. Regardless of all this importance of vitamin D, the synthesis of vitamin D from exposure to ultraviolet B radiation from the sun is the most available source of vitamin D in animals. However, intensive farming systems do not allow for sufficient sunlight exposure to sows to fulfil their vitamin D needs. As such, dietary vitamin D supplementation is required. When selecting a vitamin D metabolite to do so, it is imperative to keep the piglets in mind as well. Their feed intake is initially relatively low, so their dietary uptake of vitamin D will be limited. Yet little vitamin D could be transferred from mother to their offspring, and this mainly depends on the type of vitamin D metabolite: for example, standard vitamin D_3_ is barely transferred from sows to piglets through the placenta and/or milk [8]. However, it becomes a limiting factor in sow reproductive performance, making the choice of which vitamin D metabolite to use is even more important.

There are several forms of dietary vitamin D and metabolites that have been used in animals, with the most well-known being vitamin D_3_ (cholecalciferol). In itself, vitamin D_3_ is not metabolically active: it is first hydroxylated in the liver to 25-hydroxy-vitamin D_3_ (25OHD_3_), often referred to as calcidiol. A second hydroxylation in the kidneys results in the actual active form of vitamin D: 1,25-dihydroxy vitamin D_3_, or calcitriol. It is possible to supplement animals with any of these metabolites, although there are noticeable differences in bioavailability and practical considerations. In short, 25OHD_3_ is a preferred metabolite to supplement [9] with a stable and long life, easily absorbed, does not rely on the liver for the first hydroxylation, and does not pose any risk as it is the reserve form of vitamin D within the body [10]. Previous studies showed that dietary supplementation of 25OHD_3_ increases serum 25OHD_3_ in piglets and improves their growth performance and immune system [8,11]. Also, some studies have reported a comparative effect of different forms of vitamin D in sows and piglets [12,13]. To our knowledge, most of the available scientific research has been conducted with 25OHD_3_ produced via a synthetic pathway. To date, research on 25OHD_3_ produced via bacterial fermentation of natural substances is still limited, and thus, we aim to examine the effects of 25OHD_3_ supplementation on reproductive performance, nutrient digestibility, lameness score, milk composition, and blood profiles in sows and their offspring.

## 2. Materials and Methods

All the experimental procedures of the current study were approved by the Institutional Animal Care and Use Committee of Dankook University, Cheonan, Republic of Korea (DK-2-2327).

### 2.1. Source of 25-Hydroxy-Vitamin D_3_

The supplemented 25-hydroxy-vitamin D_3_ (Bio D^®^) was commercially obtained from Huvepharma (EOOD, Sofia, Bulgaria) and added to the sow diet at the manufacturer’s prescribed level.

### 2.2. Animals, Husbandry, and Experimental Diets

A total of 30 multiparous (Landrace × Yorkshire) sows (average parity of 3.2) were randomly allocated to one of three dietary treatments with 10 replications per treatment following a randomized complete block design. The dietary treatments were as follows: (1) CON, basal diet; (2) TRT1, CON diet plus 1114 IU 25OHD_3_/kg (13.92 µg 25OHD_3_/kg feed); and (3) TRT2, CON diet plus 2227 IU 25OHD_3_/kg (27.84 µg 25OHD_3_/kg feed). Basal diets (Table 1) were formulated according to the recommendations of the National Research Council [14] and offered to sows from 110 days of gestation and continued until weaning (21 days), whereas dam milk was the only source to piglets from birth to 21 days of weaning. From the 110th day of gestation, sows were placed in an environmentally regulated farrowing facility with a pen area of 2.1 × 0.6 m until weaning. The temperature was maintained at a maximum of 20 °C. Before farrowing, rubber mats were put down as a surface for piglets to lie on. One heat lamp was suspended above each rubber mat to keep the temperature of the newborn piglets constant at 35 °C. Sows were offered 4 kg of feed/day/sow before farrowing and 10 kg of feed/day/sow after farrowing. This feeding strategy was applied from the 3rd day after artificial insemination to 20 days after farrowing. The daily rations for sows were divided into two parts: one part was given in the morning, and the remaining half was given eight hours later. After weaning, sows were fed 3.6 kg of feed daily until ovulation. Every newborn piglet received a 1 mL iron injection within 24 h of birth. On day 5, male piglets were castrated, and all piglets were ear-tagged.

### 2.3. Sampling and Analysis

#### 2.3.1. Reproduction Performance

The sows’ body weight (BW) and body condition score (BCS) were measured before farrowing, after farrowing, and at weaning. The average daily feed intake (ADFI) was measured during the gestation and lactation period. Backfat thickness was measured at the beginning of the experimental trial, after farrowing, and at weaning using ultrasound equipment (Piglog 105, SKF Technology, Herlev, Denmark). At farrowing, the total number of piglets born, live piglets born, mummified fetuses, and the survival rate were recorded. Piglets were equally divided among sows in the same treatment group. The number of piglets born and alive at weaning, SUR (Survival rate), body weights at birth and weaning, as well as the average daily gain, were also recorded. After weaning, the sow’s estrus cycle was recorded. Sows were moved to enclosures that were near mature boars and had direct exposure to them twice a day (at 0800 and 1600 h) for estrus detection. Estrus in sows was assumed when they showed a standing reaction in response to a back-pressure test while standing next to the boar.

#### 2.3.2. Nutrient Digestibility of Sows

Apparent total tract nutrient digestibility (ATTD) of dry matter (DM), nitrogen, and energy was measured during farrowing and at weaning. Chromium oxide (Cr_2_O_3_, 0.5%), an indigestible marker, was added to the sow diet 7 days prior to fecal collection to calculate the nutrient digestibility at the end of the experiment. Chromium mixed feed samples (300 g per batch per treatment) from three batches were collected for nutrient and chromium analysis. Fecal samples were collected from each sow via rectal massage and stored at −20 °C. Feed and fecal samples were dried at 70 °C for 72 h and finely ground to be able to pass through a 1 mm screen. Samples were then analyzed for DM, nitrogen, and energy following the procedures of the Association of Official Analytical Chemists International [15]. Chromium was analyzed via a UV spectrophotometer (Optizen POP, Seoul, South Korea). To calculate the ATTD of the nutrients, we used the following formula: Digestibility = 1 − [(Nf × Cd)/(Nd × Cf)] × 100, where Nf = concentration of nutrient in excreta (% DM), Nd = concentration of nutrient in the diet, Cd = concentration of chromium in the diet, and Cf = concentration of chromium in the excreta. The gross energy was measured by measuring the heat of combustion using a Parr 6400 oxygen bomb calorimeter (Parr Instrument Co., Moline, IL, USA).

#### 2.3.3. Milk Composition of Sows

At initial and at weaning, 30 mL of milk was collected from each sow (six per treatment). The samples were sent to the laboratory to examine their proximate composition, including fat, protein, lactose, Solids Not Fat (SNF), total solids, and freezing point using a Milkoscan^TM^ Mars (FOSS, Hilleroed, Denmark). The samples were also analyzed for 25OHD_3_ content, using an RIA kit (IDS Immunodiagnostic Systems Ltd., Boldon, Tyne and Wear, UK).

#### 2.3.4. Lameness Score of Sows

Prior to and after farrowing, the lameness score was determined. The subsequent scoring scheme was employed to ascertain the lameness scores: 0 = Sow walks freely, employing both feet and all four limbs equally; 1 = Sow moves weight away from the injured limb when standing, but does not exhibit any lameness or limping when walking; 2 = Sow clearly shifts weight away from the injured limb when standing, and exhibits adaptive behavior (head, accelerated stride on affected limb) when walking; 3 = The sow exhibits reluctance to stand and/or walk, a noticeable limp, and adaptive walking habits; 4 = The sow does not bear any weight on the injured limb when standing or moving around.

#### 2.3.5. Blood Profile of Sow and Piglets

Blood samples were collected from each sow in non-anticoagulant tubes at day 75 of gestation and at weaning (5 mL/sow). Similarly, 5 piglets per pen were selected, and blood samples were collected in a vacuum tube (5 mL/piglet; Becton Dickson Vacutainer Systems, Franklin Lakes, NJ, USA). Serum calcium, phosphorus, and 25OHD_3_ were measured using an automatic biochemical analyzer (HITACHI 747, Tokyo, Japan). For immunoglobulin G (IgG), samples were analyzed using a pig IgG ELISA kit according to the manufacturer’s instructions (ELISA Starter Accessory Package, Pig IgG ELISA Quantitation Kit; Bethyl, Montgomery, TX, USA).

### 2.4. Statistical Analysis

To analyze the results on sow performance, each sow was considered to be an experimental unit. For piglet performance, each farrowing pen was taken as the experimental unit. Backfat thickness was counted as a covariate for backfat thickness change during lactation. Piglet birth weight was used as a covariate for the growth performance of piglets. Data were analyzed using a general linear model (SAS 9.0 version, SAS Inst. Inc., Cary, NC, USA). A Tukey’s multiple range test was performed. A probability value of *p* < 0.05 was considered to indicate a significant difference, while *p* < 0.10 was considered indicative of a tendency.

## 3. Results

### 3.1. Reproduction Performance in Sows

The reproductive performance and body condition of sows fed 25OHD_3_ supplementation are presented in Table 2. The total number of piglets born, number of stillbirths, mummified fetuses, and the number of piglets born alive were not affected by dietary treatment (*p* > 0.05). Similarly, the survival rate of piglets showed no significant differences among groups. The BW, BWD, BFT, and BFTD during gestation and lactation were not significantly influenced by treatment (*p* > 0.05). Likewise, BCS and ADFI during gestation and lactation remain similar among all groups (*p* > 0.05). However, sows fed 25OHD_3_ supplementation exhibited shorter farrowing times compared with the CON.

### 3.2. Growth Performance in Piglets

Sows in the TRT2 group produced a higher number of piglets both at birth and at weaning compared with the CON group (Table 3). However, the survival rate of piglets remains. Also, piglet BW at birth showed no significant difference among groups. However, piglets from TRT1 and TRT2 sows had significantly higher (*p* < 0.05) weaning weights than the CON group. Similarly, the ADG of piglets was greater (*p* < 0.05) in the TRT1 and TRT2 groups compared with the CON group.

### 3.3. Lameness Score in Sows

Lameness score of sows in the different groups is shown in Table 4. There was no significant difference observed in the lameness score during the entire study period (*p* > 0.05).

### 3.4. Nutrient Digestibility in Sows

The effects of dietary 25OHD_3_ supplementation on the nutrient digestibility of sows are presented in Table 5. No significant differences were observed among treatments for the digestibility of nutrients throughout the experimental period (*p* > 0.05).

### 3.5. Milk Contents in Sows

The effects of dietary 25OHD_3_ supplementation on colostrum and milk composition of sows are presented in Table 6. In colostrum, the protein content was significantly higher (*p* < 0.05) in sows fed 25OHD_3_ compared with the CON group. However, the concentrations of fat, lactose, solids not fat, and total solids were not affected by dietary treatments (*p* > 0.05). The concentration of 25OHD_3_ in colostrum increased (*p* < 0.05) markedly with supplementation, being higher in the TRT1 and 2 groups than in CON. Similar to colostrum milk, during weaning, the 25OHD_3_ concentration in milk was significantly higher in the TRT1 and 2 groups than in the CON group.

### 3.6. Blood Profile in Sows

The effects of dietary 25OHD_3_ supplementation on blood metabolites of sows are shown in Table 7. At day 75 of gestation, serum calcium, phosphorus, and immunoglobulin G (IgG) concentrations were not significantly affected by dietary treatments (*p* > 0.05). However, the serum 25OHD_3_ concentration was markedly higher (*p* < 0.05) in sows fed 25OHD_3_ compared with the CON. Similarly, at weaning, no significant differences were observed among treatments in serum calcium, phosphorus, or IgG levels (*p* > 0.05). However, serum 25OHD_3_ concentration remained significantly greater (*p* > 0.05) in the TRT1 and 2 groups than in the CON group.

### 3.7. Blood Profile in Piglets

Table 8 lists the impact of dietary 25OHD_3_ on the blood profile of piglets. Significant increases in serum 25OHD_3_ concentrations were observed in the treatment groups (*p* = 0.015). Similarly, IgG concentrations were also significantly higher in these groups (*p* = 0.016). Other blood parameters improved numerically with increasing doses of 25OHD_3_.

## 4. Discussion

A dietary supplementation with 25OHD_3_ showed significant effects in both sows and their offspring. Beyond the numerical improvements in reproductive parameters, the most notable outcome was a significant reduction in farrowing time, which aligns with previous studies reporting similar beneficial effects of 25OHD_3_ supplementation on parturition performance [5]. Herein, we proposed that an additional 25OHD_3_ might have supported uterine contractions during farrowing, either directly or indirectly through enhanced calcium availability essential for proper muscle function, but the exact mechanism behind reducing farrowing time is currently unknown and thus warrants further research. Consistent with the more efficient farrowing process, 25OHD_3_ supplementation in the sow diet also resulted in significantly improved numbers of piglets at birth and weaning stage, and increased offspring BW at weaning. This improvement was further supported by the significantly greater ADG of piglets born to 25OHD_3_-supplemented sows, and these findings were aligned with the previous research [16,17]. The positive findings observed in the trial were mainly attributed to vitamin D supplementation during early life, which is known to positively influence growth and technical performance [18,19,20]. As noted earlier, standard vitamin D_3_ is poorly transferred from the sow to the piglet, whether through the placenta or milk. Since piglets in this study were not provided with creep feed, all their vitamin D intake depended on colostrum and milk. The elevated 25OHD_3_ concentrations in milk from supplemented sows suggest that piglets received a higher amount of vitamin D, which likely supported their physiological needs and contributed to improved growth performance. Additional mechanisms may also be involved; for instance, Van Hemelrijck et al. [19] reported that circulating 25OHD_3_ concentrations can promote the secretion of growth-related hormones. Moreover, the inclusion of 25OHD_3_ supplementation in the sow diet had a positive influence on milk composition. This observation is consistent with Zhou et al. [5], who demonstrated that maternal 25OHD_3_ supplementation increased milk protein content. The elevated protein levels in milk from supplemented sows may have provided additional nutrients essential for neonatal growth and tissue development, thereby contributing to the superior BW and growth rate. Improved milk quality not only enhances the nutritional value available to suckling piglets but may also reflect the improved metabolic and hormonal status of the sow under 25OHD_3_ supplementation. Collectively, these effects underscore the pivotal role of maternal vitamin D status in shaping milk composition and promoting optimal postnatal development in piglets. The ATTD of sows was not affected by 25OHD_3_ supplementation, which aligns with the findings of Upadhaya et al. [16] and Flohr et al. [21]. One possible explanation for this lack of effect is that the sows were already fed diets that met or exceeded their nutrient requirements, particularly for energy, protein, calcium, and phosphorus. Under these conditions, an additional 25OHD_3_ may not further improve nutrient absorption, as the digestive and metabolic systems are likely already functioning near their maximal capacity. Furthermore, research on the effects of 25OHD_3_ supplementation in sows remains limited, and thus sufficient comparison could not be made. Regarding blood profiles, serum 25OHD_3_ concentrations were significantly higher in both sows and piglets from the treatment groups compared with the control group. As circulating 25OHD_3_ is a recognized biomarker of physiological vitamin D status [22,23], these results confirm that the 25OHD_3_ of fermentative origin used in this study is an effective dietary supplement for improving vitamin D status. The findings also emphasize the high bioavailability and absorption efficiency of naturally derived 25OHD_3_ compared with standard vitamin D_3_, consistent with earlier reports showing increased circulating 25OHD_3_ levels following dietary supplementation [24]. In addition to improvements in serum 25OHD_3_, other blood parameters were also positively affected. Notably, IgG concentrations were higher in both sows and piglets from the 25OHD_3_-supplemented groups. As IgG is the principal circulating antibody essential for humoral immunity, its role in infection prevention is well established [4]. Previous research has suggested that various vitamin D metabolites can modulate immune responses [25,26,27]. A human study also demonstrated a strong correlation between serum 25OHD_3_ and IgG levels [28], while a study in piglets showed higher serum IgG concentrations following 25OHD_3_ supplementation compared to regular vitamin D_3_ [29], further supporting the immunomodulatory benefits of 25OHD_3_ in swine.

## 5. Conclusions

The inclusion of dietary supplementation of 25OHD_3_ in the sow diet significantly reduces the farrowing time and growth and immune resilience in their offspring. These findings highlight 25OHD_3_ as a superior alternative to conventional vitamin D_3_, with clear implications for improving swine productivity, offspring viability, and overall herd health, providing a practical strategy to optimize intensive swine production systems.

## Figures and Tables

**Table 1:** Composition of the basal diet (sow, as-fed basis).

Ingredient, %	Gestation	Lactation
Corn	52.17	41.93
Wheat	21.50	23.00
Wheat bran	7.01	8.31
Soybean meal, 48%	3.49	4.48
Dehulled Soybean meal	6.93	12.96
Molasses	2.00	2.00
Soybean oil	3.46	3.40
MCP	0.95	1.20
Limestone	1.25	1.18
MgO	0.02	0.02
Salt	0.50	0.50
Threonine (99%)	0.10	0.17
Methionine (99%)	-	0.02
L-lysine (78%)	0.12	0.31
Vit/Mineral premix ^1^	0.40	0.40
Choline (25%)	0.10	0.12
Total	100.00	100.00
Calculated value		
CP, %	13.00	16.50
ME, kcal/kg	3300	3300
FAT, %	5.94	5.71
Ca, %	0.72	0.76
P, %	0.55	0.65
LYS, %	0.62	0.96
THR, %	0.16	0.65
MET, %	0.48	0.26

^1^ Provided per kilogram of complete diet: vitamin A, 12,100 IU; vitamin D_3_, 2000 IU; vitamin E, 48 IU; vitamin K3, 1.5 mg; riboflavin, 6 mg; niacin, 40 mg; d-pantothenic, 17 mg; biotin, 0.2 mg; folic acid, 2 mg; choline, 166 mg; vitamin B6, 2 mg; and vitamin B12, 28 lg. Fe (as FeSO_4_7H_2_O), 90 mg; Cu (as CuSO_4_5H_2_O), 15 mg; Zn (as ZnSO_4_), 50 mg; Mn (as MnO_2_), 54 mg; I (as KI), 0.99 mg; and Se (as Na_2_SeO_3_5H_2_O), 0.25 mg.

**Table 2:** The effect of dietary 25-hydroxy-vitamin D_3_ supplementation on reproduction performance in lactating sows ^1^.

Items	CON	TRT1	TRT2	SEM ^2^	*p*-Value ^3^
Parity	3.2	3.2	3.2	0.3	1.000
Litter size					
Total birth, head	12.5	12.4	12.7	0.7	0.860
Mummification, head	0.3	0.2	0.0	0.1	0.257
Stillbirth, head	0.4	0.2	0.4	0.2	0.630
Total alive, head	11.8	12.0	12.3	0.7	0.636
SUR1 ^4^, %	94.59	96.69	97.27	1.92	0.141
Body weight, kg					
Initial	179.1	178.9	179.7	4.0	0.940
B. Farrowing	207.8	208.6	210.2	4.5	0.806
A. Farrowing	188.3	188.2	189.3	4.3	0.914
Weaning	172.9	173.5	175.0	4.1	0.809
BWD 1 ^5^	28.8	29.7	30.5	0.6	0.156
BWD 2 ^5^	19.5	20.4	20.9	0.7	0.277
BWD 3 ^5^	15.4	14.7	14.2	0.8	0.350
Backfat thickness, mm					
Initial	16.3	16.4	16.4	0.3	0.872
B. Farrowing	19.4	19.8	19.9	0.5	0.511
A. Farrowing	17.7	17.9	18.0	0.4	0.592
Weaning	15.5	16.0	16.2	0.3	0.155
BFTD 1 ^6^	3.1	3.4	3.5	0.3	0.360
BFTD 2 ^6^	1.7	1.9	1.9	0.3	0.632
BFTD 3 ^6^	2.2	1.9	1.8	0.2	0.276
Body condition score					
Initial	2.5	2.5	2.5	0.1	1.000
B. Farrowing	3.2	3.5	3.5	0.1	0.171
A. Farrowing	3.0	3.1	3.2	0.2	0.663
Weaning	2.4	2.6	2.6	0.1	0.470
ADFI, kg					
Gestation	2.47	2.48	2.48	0.01	0.920
Lactation	7.23	7.27	7.30	0.05	0.629
Estrus interval, d	5.1	4.9	4.8	0.5	0.880
Farrowing time, min	213.0 ^a^	165.0 ^b^	157.0 ^b^	8.1	<0.001

^1^ Abbreviation: CON, Basal diet; TRT1, Basal diet + 1114 25OHD_3_; TRT2, Basal diet + 2227 25OHD_3_, BWD, body weight difference; BFTD, backfat thickness difference. ^2^ Standard error of means. ^3^ Means in the same row with different superscripts differ significantly (*p* < 0.05). ^4^ SUR1: Survival rate of the number of alive pigs per the number of total born pigs. ^5^ BWD 1: Initial to Before farrowing; BWD 2: Before farrowing to after farrowing; BWD 3: After farrowing to weaning. ^6^ BFTD 1: Initial to Before farrowing; BFTD 2: Before farrowing to after farrowing; BFTD 3: After farrowing to weaning.

**Table 3:** The effect of dietary 25-hydroxy-vitamin D_3_ supplementation on growth performance in suckling piglets ^1^.

Items	CON	TRT1	TRT2	SEM ^2^	*p*-Value ^3^
Litter Size					
At birth	11.8 ^b^	12.^ab^	12.3^a^	0.1	0.013
At weaning	11.3 ^b^	11.7 ^ab^	12.1 ^a^	0.2	0.015
SUR2 ^4^, %	95.76	97.50	98.33	1.16	0.303
Body weight, kg					
Birth weight	1.21	1.28	1.30	0.03	0.109
Weaning	5.99 ^b^	6.41 ^a^	6.55 ^a^	0.12	0.008
Average daily gain, g	227 ^b^	244 ^a^	250 ^a^	5	0.007

^1^ Abbreviation: CON, Basal diet; TRT1, Basal diet + 1114 25OHD_3_; TRT2, Basal diet + 2227 25OHD_3_. ^2^ Standard error of means. ^3^ Means in the same row with different superscripts differ significantly (*p* < 0.05). ^4^ SUR2: Survival rate during lactation.

**Table 4:** The effect of dietary 25-hydroxy-vitamin D_3_ supplementation on lameness score in lactating sow ^1^.

Items	CON	TRT1	TRT2	SEM ^2^	*p*-Value ^3^
Lameness score ^4^					
Initial	0.7	0.7	0.8	0.2	0.893
B. Farrowing	1.0	0.9	0.8	0.2	0.708
A. Farrowing	1.2	1.0	1.0	0.2	0.716
Weaning	1.0	0.9	0.9	0.2	0.927

^1^ Abbreviation: CON, Basal diet; TRT1, Basal diet + 1114 25OHD_3_; TRT2, Basal diet + 2227 25OHD_3_. ^2^ Standard error of means. ^3^ Level of significance (*p* < 0.05). ^4^ Lameness scores were determined using the following lameness scoring system: 0 Sow moves freely and uses all 4 limbs and feet evenly.

**Table 5:** The effect of dietary 25-hydroxy-vitamin D_3_ supplementation on Nutrient digestibility in lactating sows ^1^.

Items	CON	TRT1	TRT2	SEM ^2^	*p*-Value ^3^
B. Farrowing					
Dry matter	64.10	64.27	64.31	0.51	0.953
Nitrogen	60.74	60.93	61.13	0.52	0.874
Energy	63.16	63.21	63.33	0.46	0.966
Weaning					
Dry matter	63.76	63.03	64.15	0.55	0.368
Nitrogen	59.98	60.10	60.33	0.54	0.899
Energy	62.57	62.69	62.82	0.55	0.947

^1^ Abbreviation: CON, Basal diet; TRT1, Basal diet + 1114 25OHD_3_; TRT2, Basal diet + 2227 25OHD_3_. ^2^ Standard error of means. ^3^ Level of significance (*p* < 0.05).

**Table 6:** The effect of dietary 25-hydroxy-vitamin D_3_ supplementation on milk profile in lactating sow ^1^.

Items	CON	TRT1	TRT2	SEM ^2^	*p*-Value ^3^
Colostrum					
Fat, %	3.65	3.78	3.90	0.08	0.141
Protein, %	14.19 ^b^	14.98 ^a^	15.23 ^a^	0.58	0.035
Lactose, %	2.64	2.80	2.88	0.15	0.537
Solids Not Fat, %	19.59	19.78	19.95	0.43	0.848
Total Solids, %	22.50	22.89	23.06	0.96	0.915
25OHD_3_, ng/g	0.392 ^b^	0.796 ^a^	0.864 ^a^	0.021	<0.001
Frozen Point, °C	−0.55	−0.55	−0.55	-	
Weaning					
Fat, %	8.72	8.82	8.91	0.11	0.514
Protein, %	4.63	4.87	5.03	0.17	0.298
Lactose, %	5.37	5.54	5.71	0.15	0.350
Solids Not Fat, %	10.68	10.82	11.01	0.11	0.172
Total Solids, %	20.44	20.69	20.87	1.14	0.964
25OHD_3_, ng/g	0.522 ^b^	0.986 ^a^	1.113 ^a^	0.037	<0.001
Frozen Point, °C	−0.57	−0.57	−0.57	-	

^1^ Abbreviation: CON, Basal diet; TRT1, Basal diet + 1114 25OHD_3_; TRT2, Basal diet + 2227 25OHD_3_. ^2^ Standard error of means. ^3^ Means in the same row with different superscripts differ significantly (*p* < 0.05).

**Table 7:** The effect of dietary 25-hydroxy-vitamin D_3_ supplementation on the blood profile in lactating sows ^1^.

Items	CON	TRT1	TRT2	SEM ^2^	*p*-Value ^3^
Day 75					
Calcium, mg/dL	9.15	9.31	9.54	0.33	0.705
Phosphorus, mg/dL	4.84	5.00	5.31	0.16	0.143
IgG, mg/dL	377.7	386.7	395.4	12.6	0.617
25OHD_3_, ng/mL	37.73 ^b^	65.39 ^a^	69.60 ^a^	2.45	<0.001
Weaning					
Calcium, mg/dL	9.28	9.60	9.87	0.30	0.389
Phosphorus, mg/dL	5.01	5.43	5.76	0.28	0.157
IgG, mg/dL	348.9	364.4	371.7	10.9	0.341
25OHD_3_, ng/mL	33.34 ^b^	54.36 ^a^	60.83 ^a^	2.23	<0.001

^1^ Abbreviation: CON, Basal diet; TRT1, Basal diet + 1114 25OHD_3_; TRT2, Basal diet + 2227 25OHD_3_. ^2^ Standard error of means. ^3^ Means in the same row with different superscripts differ significantly (*p* < 0.05).

**Table 8:** The effect of dietary 25-hydroxy-vitamin D_3_ supplementation on blood profile in suckling piglets ^1^.

Items	CON	TRT1	TRT2	SEM ^2^	*p*-Value ^3^
Weaning					
Calcium, mg/dL	10.12	10.41	10.78	0.39	0.498
Phosphorus, mg/dL	9.14	9.50	9.79	0.34	0.432
IgG, mg/dL	226.8 ^b^	255.1 ^a^	258.9 ^a^	10.9	0.016
25OHD_3_, ng/mL	6.54 ^b^	8.16 ^a^	8.42 ^a^	0.84	0.015

^1^ Abbreviation: CON, Basal diet; TRT1, Basal diet + 1114 25OHD_3_; TRT2, Basal diet + 2227 25OHD_3_. ^2^ Standard error of means. ^3^ Means in the same row with different superscripts differ significantly (*p* < 0.05).

## Data Availability

The data presented in this study are available on request from the corresponding author.

## References

[1] Patterson J., Foxcroft G. (2019). Gilt Management for Fertility and Longevity. Animals.

[2] Yatabe Y., Iida R., Piñeiro C., Koketsu Y. (2019). Recurrence patterns and lifetime performance of parity 1 sows in breeding herds with different weaning-to-first-mating intervals. Porc. Health Manag..

[3] Dicken C.L., Israel D.D., Davis J.B., Sun Y., Shu J., Hardin J., Neal-Perry G. (2012). Peripubertal vitamin D_3_ deficiency delays puberty and disrupts the estrous cycle in adult female mice. Biol. Reprod..

[4] Belenchia A.M., Jones K.L., Will M., Beversdorf D.Q., Vieira-Potter V., Rosenfeld C.S., Peterson C.A. (2018). Maternal vitamin D deficiency during pregnancy affects expression of adipogenic-regulating genes peroxisome proliferator-activated receptor gamma (PPARgamma) and vitamin D receptor (VDR) in lean male mice offspring. Eur. J. Nutr..

[5] Zhang J.Y., Bae J.E., Jeong Y.J., Kim I.H. (2017). Impact of 25-hydroxyvitamin D on productive performance of gestating sows. Korean J. Agric. Sci..

[6] Tousignant S.J., Henry S.C., Rovira A., Morrison R.B. (2013). Effect of oral vitamin D_3_ supplementation on growth and serum 25-hydroxy vitamin D levels of pigs up to 7 weeks of age. J. Swine Health Prod..

[7] O’Brien M.A., Jackson M.W. (2012). Vitamin D and the immune system: Beyond rickets. Vet. J..

[8] Thayer M.T., Nelssen J.L., Langemeier A.J., Morton J.M., Gonzalez J.M., Kruger S.R., Ou Z., Makowski A.J., Bergstrom J.R. (2019). The effects of maternal dietary supplementation of cholecalciferol (vitamin D_3_) and 25(OH)D_3_ on sow and progeny performance1. Transl. Anim. Sci..

[9] Kolp E., Wilkens M.R., Pendl W., Eichenberger B., Liesegang A. (2017). Vitamin D metabolism in growing pigs: Influence of UVB irradiation and dietary vitamin D supply on calcium homeostasis, its regulation and bone metabolism. J. Anim. Physiol. Anim. Nutr..

[10] Zhang L., Piao X. (2021). Use of 25-hydroxyvitamin D_3_ in diets for sows: A review. Anim. Nutr..

[11] Zhou H., Chen Y., Lv G., Zhuo Y., Lin Y., Feng B., Fang Z., Che L., Li J., Xu S. (2016). Improving maternal vitamin D status promotes prenatal and postnatal skeletal muscle development of pig offspring. Nutrition.

[12] Lauridsen C., Halekoh U., Larsen T., Jensen S.K. (2010). Reproductive performance and bone status markers of gilts and lactating sows supplemented with two different forms of vitamin D1. J. Anim. Sci..

[13] Upadhaya S.D., Chung T.K., Jung Y.J., Kim I.H. (2022). Dietary 25(OH)D_3_ supplementation to gestating and lactating sows and their progeny affects growth performance, carcass characteristics, blood profiles and myogenic regulatory factor-related gene expression in wean-finish pigs. Anim. Biosci..

[14] NRC—National Research Council (2012). Nutrient Requirements of Swine.

[15] AOAC (2020). Official Methods of Analysis.

[16] Upadhaya S.D., Jung Y.J., Kim Y.M., Chung T.K., Kim I.H. (2021). Effects of dietary supplementation with 25-OH-D3 during gestation and lactation on reproduction, sow characteristics and piglet performance to weaning. Anim. Feed. Sci. Technol..

[17] Weber G.M., Witschi A.-K.M., Wenk C., Martens H. (2014). Triennial Growth Symposium—Effects of dietary 25-hydroxycholecalciferol and cholecalciferol on blood vitamin D and mineral status, bone turnover, milk composition, and reproductive performance of sows. J. Anim. Sci..

[18] Lucas R.M., Ponsonby A.L., Pasco J.A., Morley R. (2008). Future health implications of prenatal and early-life vitamin D status. Nutr. Rev..

[19] Van Hemelrijck M., Shanmugalingam T., Bosco C., Wulaningsih W., Rohrmann S. (2015). The association between circulating IGF1, IGFBP3, and calcium: Results from NHANES III. Endocr. Connect..

[20] Zhou H., Chen Y., Zhuo Y., Lv G., Lin Y., Feng B., Fang Z., Che L., Li J., Xu S. (2017). Effects of 25—hydroxycholecalciferol supplementation in maternal diets on milk quality and serum bone status markers of sows and bone quality of piglets. Anim. Sc. J..

[21] Flohr J.R., Woodworth J.C., Bergstrom J.R., Tokach M.D., Dritz S.S., Goodband R.D., DeRouchey J.M. (2016). Evaluating the impact of maternal vitamin D supplementation on sow performance: II. Subsequent growth performance and carcass characteristics of growing pigs. J. Anim. Sci..

[22] Lee S.A., Torres-Mendoza L.J., Stein H.H. (2022). Effects of 25-hydroxycholecalciferol (25-OH-D_3_) and 1-hydroxycholecalciferol (1-OH-D_3_) on serum bone biomarkers and calcium and phosphorus balance and concentrations of energy in diets without or with microbial phytase fed to sows in late gestation. J. Anim. Sci..

[23] Palacios C., Gonzalez L. (2014). Is vitamin D deficiency a major global public health problem?. J. Steroid Biochem. Mol. Biol..

[24] Coffey J.D., Hines E.A., Starkey J.D., Starkey C.W., Chung T.K. (2012). Feeding 25-hydroxycholecalciferol improves gilt reproductive performance and fetal vitamin D status. J. Anim. Sci..

[25] Bikle D.D. (2009). Vitamin D and immune function: Understanding common pathways. Curr. Osteoporos. Rep..

[26] Hashim Z.R., Qasim Q.A., ALabbood M.H. (2022). The association of serum calcium and vitamin D with insulin resistance and beta-cell dysfunction among people with type 2 diabetes. Arch. Razi Inst..

[27] Lalor M.K., Floyd S., Gorak-Stolinska P., Weir R.E., Blitz R., Branson K., Fine P.E., Dockrell H.M. (2011). BCG Vaccination: A Role for Vitamin D?. PLoS ONE.

[28] Pincikova T., Nilsson K., Moen I.E., Karpati F., Fluge G., Hollsing A., Knudsen P.K., Lindblad A., Mared L., Pressler T. (2011). Inverse relation between vitamin D and serum total immunoglobulin G in the Scandinavian Cystic Fibrosis Nutritional Study. Eur. J. Clin. Nutr..

[29] Zhang L., Yang M., Piao X. (2022). Effects of 25-hydroxyvitamin D_3_ on growth performance, serum parameters, fecal microbiota, and metabolites in weaned piglets fed diets with low calcium and phosphorus. J. Sci. Food Agric..

